# Mechanics of liquid crystal inclusions in soft matrices

**DOI:** 10.1016/j.jmps.2025.106070

**Published:** 2025-04

**Authors:** Yifei Bai, Laurence Brassart

**Affiliations:** Department of Engineering Science, University of Oxford, Oxford OX1 3PJ, United Kingdom

**Keywords:** Nematic liquid crystal, Hyperelasticity, Anisotropic surface tension, Elasto-capillarity, Composite

## Abstract

The mechanical behaviour of composites of liquid crystal inclusions embedded in soft matrices involves a complex interplay between the elasticity of the matrix, the surface elasticity of the interfaces, and the reorientation of the liquid crystal molecules. Directors of the (nematic) liquid crystal tend to be aligned in the bulk, but may ”anchor” along the interface. In addition, the interface deforms according to the bulk deformation, while trying to minimise the surface area. In this paper, we present a continuum theory for an incompressible hyperelastic matrix containing nematic liquid crystal inclusions. The elastic energy of the inclusions, attributed to the distortion of the director field, is described using Landau–de Gennes theory. The matrix is described as an incompressible neo-Hookean solid. Anchoring effects at the inclusion–matrix interface are described through anisotropic surface tension. The model is implemented numerically using the FEniCSx finite element code. Through parametric study, we investigate the impact of energy competitions on the macroscopic and inclusion responses. Similar to the case of liquid inclusions, composites containing liquid crystal inclusions can be stiffer or softer than the matrix, depending on the value of the elasto-capillary number. The softening or stiffening effect is further affected by the distortional energy of the inclusion and the anchoring strength of the interface. Conversely, applied mechanical loads can reorient the director field. In particular, we show that stress-induced reorientation is significant when the dimensionless volume of the inclusion is large, involving alignment of the directors under tension, and disorientation under compression. The proposed theory and new physical insights could be useful for the design of smart stimuli-responsive materials.

## Highlights

• Continuum theory for nematic LC inclusions in elastic matrices.
• Anisotropic surface tension couples matrix deformation and LC ordering.
• LC inclusions can reinforce soft matrices.
• Reinforcing effect enhanced by larger anchoring strength.
• Mechanical loads can reorient the director field in the inclusion.

## 1 Introduction

**Fig. 1:**
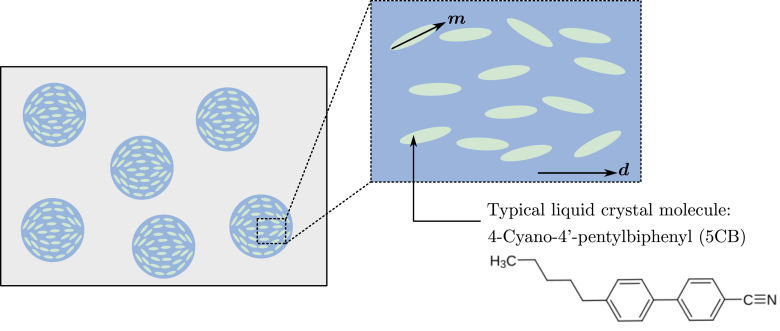
Schematic of a polymer dispersed liquid crystal composite. The liquid crystal phase consists of elongated molecules, such as 5CB.

Liquid crystal is a state of matter that can flow like a liquid, but possesses certain orientational ordering in space due to the geometric anisotropy of the molecules (de Gennes and Prost, 1993). Due to its mobility, it can respond sensitively to external stimulus like temperature and electric or magnetic fields, and presents orientational-specific behaviour. In applications, liquid crystal materials are usually combined with stiffer materials to achieve suitable mechanical properties while preserving the mobility of the liquid crystal phase. For example, Polymer-Dispersed Liquid Crystal (PDLC) consisting of liquid crystal inclusions dispersed in a polymer matrix are commonly used in applications such as smart windows (Lu et al., 2024, Ghosh, 2023, Kamal et al., 2022), photovoltaic devices (Khalid et al., 2021) and smart lenses (Ren et al., 2005, Zhang et al., 2020). Fig. 1 illustrates the microstructure of a PDLC composite. In the liquid crystal phase, the average orientation of the molecules at a material point is described by the director vector d. In the applications mentioned above, the in-service deformation is usually very small, so that the inclusion shape barely changes. Also, the resistance of the matrix to deformation is much larger than that of the interface or the liquid crystal phase, so that the coupling between elastic deformation of the matrix and liquid crystal ordering can be neglected.

In recent years, liquid crystal inclusions embedded in soft matrices have been developed for applications such as sensors, wearable devices and all-solid electrolytes (Song et al., 2017, Zhang et al., 2023, Froyen and Schenning, 2023, Hadjichristov, 2023), combining the stimuli-responsiveness and optical properties of the liquid crystal and the extensibility of the soft polymeric matrix. Liquid crystal inclusions in soft matrices are also observed in nature. For example, filamentous phages produced by bacteria can self-assemble into liquid crystalline droplets encapsulating the bacteria to protect them from antibiotics (Tarafder et al., 2020). In these systems, a coupling between the elastic deformation of the matrix and liquid crystal ordering is expected. For example, Balenko et al. (2021) studied the mechano-optical properties of cholesteric liquid crystal inclusions embedded in polyurethane (PU). These authors found that liquid crystal molecules reorient under an applied deformation, resulting in colour change. In their more recent study, Balenko et al. (2023) also found that the sensitivity of deformation-induced colour change depends not only on the magnitude of the deformation, but also on the stiffness of the matrix. We interpret this phenomenon in the following way. When the elastic matrix is deformed, the displacement field at the matrix/inclusion interface changes the shape of the inclusion. This shape change of the interface in turn reorients the liquid crystal molecules in the inclusions through the anchoring of liquid crystal molecules at the interface. However, a theoretical model describing these coupled phenomena is lacking.

Our problem is closely related to the problem of a soft matrix containing liquid inclusions. Style et al. (2015a) have shown that a soft silicone matrix (shear modulus G=1.7kPa) containing ionic liquid inclusions is stiffened by liquid inclusions when the inclusions are small (≤6μm). This effect is attributed to the coupling between matrix elasticity and surface energy of the interface (elasto-capillarity), so that small inclusions are resistant to deformations that increase their surface area. Elasto-capillary problems of isotropic liquids have been extensively studied since the foundational work by Gurtin and Murdoch (1975), followed by theoretical and numerical studies (Duan et al., 2005, Style et al., 2015a, Henann and Bertoldi, 2014, Wang and Henann, 2016, Krichen et al., 2019, Ghosh and Lopez-Pamies, 2022, Zhu and Liu, 2023). Generalised theoretical and computational frameworks have also been proposed to deal with anisotropic surface energy (Steinmann, 2008, Javili and Steinmann, 2010). A typical model of anisotropic interfacial energy is the Rapini-Papoular energy model (Rapini and Papoular, 1969), where the interface not only favours a minimal surface area, but also penalises the deviation of the surface tangential direction from a preferred direction (sometimes referred to as “easy axis”).

The coupling between liquid crystal ordering and interface deformation has also received considerable attention. The orientational ordering of liquid crystals can be described by phenomenological continuum models like Ericksen–Leslie (Ericksen, 1962, Leslie, 1979) and Oseen-Frank (Oseen, 1933, Frank, 1958) director theories, and Landau–de Gennes (de Gennes and Prost, 1993) order parameter tensor theories, where the order parameter tensor describes not only the average orientation (director), but also the ordering of the molecules. For uniaxial nematic liquid crystals, the director field far from interfaces tends to be uniform. At the interface, the liquid crystal molecules tend to align parallel to the surface, i.e. the “easy axis” coincides with the director. This so-called “anchoring effect” introduces a gradient in the director field between the interface and the far field, which is accompanied by an increase in elastic free energy. The competition between the distortional elastic energy of the liquid crystal and the interfacial energy ultimately determines the shape of the liquid crystal inclusion. Problems of (nematic) liquid crystal inclusions with free surfaces have long been studied (Casagrande et al., 1987, Virga, 2019). Kaznacheev et al. (2002) and Prinsen and van der Schoot, 2003, Prinsen and van der Schoot, 2004 proposed semi-analytical, approximate approaches by treating the surface and director fields as revolving circular arcs with different radii. Numerical simulations including Monte Carlo simulations (Bates, 2003) and finite element simulations (DeBenedictis and Atherton, 2016, Adler et al., 2023) have also been conducted to solve these problems.

Although the interaction between liquid crystal orientational ordering and interface, and between the interface and matrix elasticity have been studied separately in different contexts, the coupling of these two problems has not been investigated. In this paper, we propose a continuum mechanics model taking into account all the physics described above. We look at a model system consisting of an incompressible hyperelastic matrix containing nematic liquid crystal inclusions modelled by Landau–de Gennes theory. We follow Steinmann, 2008, Javili and Steinmann, 2010 on the modelling of a Rapini-Papoular type interfacial energy (Rapini and Papoular, 1969, Fournier and Galatola, 2005) to model anchoring. Through a variational formulation of the problem based on the principle of minimum potential energy, we obtain the weak and strong forms of the governing equations. The proposed model is then implemented in the FEniCSx finite element code using a mixed formulation. We first validate our computational model by comparing its predictions to experimental data, as well as to previous theoretical estimates for the free liquid crystal droplet problem (Virga, 2019). We then investigate the effective and microscopic properties of composites made of liquid crystal inclusions embedded in a soft matrix.

The paper is organised as follows. In Section 2, we present the continuum framework, including kinematics (Section 2.1), free energy functions (Section 2.2), and variational formulation (Section 2.3). In Section 3, we detail how the theory is implemented into a finite element code, where in Section 3.1 we define the geometry and meshes and in Section 3.2 we present the mixed finite element formulation of the problem. We also identify in Sections 3.3, 3.4 dimensionless parameters that are of interest in this study. In Section 4, we present numerical results from finite element simulations, including the determination of the equilibrium shape of a free liquid crystal droplet in a fluidic environment (Section 4.1), and the parametric study on the effect of dimensionless parameters on the effective mechanical properties of the composite as well as the coupling effects between external loading and the liquid crystal orientational ordering (Section 4.2). Concluding remarks are given in Section 5.

## 2 Theory

### 2.1 Kinematics

**Fig. 2:**
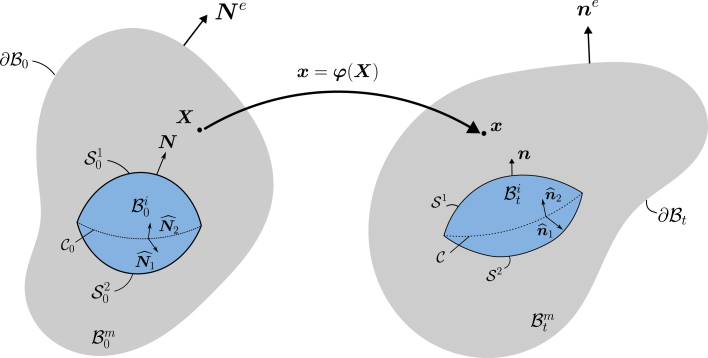
Schematic of the initial and current configurations for a liquid crystal inclusion embedded in a matrix.

We consider a body made of an elastic matrix and a liquid crystal inclusion which takes the initial, reference configuration $B_{0}$ at time t=0, see Fig. 2. The subdomains occupied by the matrix and inclusion are denoted by $B_{0}^{m}$ and $B_{0}^{i}$, respectively, and are such that $B_{0}=B_{0}^{m}\cup B_{0}^{i}$. The external boundary of the body is denoted by $\partial B_{0}$, and its outward unit normal is $N^{e}$. The interface between the matrix and the inclusion is denoted by $S_{0}$, and we write N its unit normal pointing towards the matrix. For the sake of illustration, the interface is further decomposed into two subsurfaces: $S=S_{0}^{1}\cup S_{0}^{2}$, bounded by the closed curve $C_{0}=\partial S_{0}^{1}=\partial S_{0}^{2}$ (Henann and Bertoldi, 2014). We define N^i as the unit vector normal to the curve $C_{0}$, and tangential to the subsurface $S_{0}^{i},\;\left(i=1,2\right)$. The current configuration of the body at time t>0 is denoted by $B_{t}=B_{t}^{m}\cup B_{t}^{i}$, with $B_{t}^{m}$ and $B_{t}^{i}$ the matrix and inclusion subdomains in the current configuration. The external boundary in the current configuration is written as $\partial B_{t}$ and its outward unit normal is denoted by $n^{e}$. The matrix-inclusion interface in the current configuration is written as $S_{t}=S_{t}^{1}\cup S_{t}^{2}$, with $C_{t}=\partial S_{t}^{1}=\partial S_{t}^{2}$. The unit normal to the interface pointing towards the matrix in the current configuration is written n. The curve unit normals in the current configuration are written n^1 and n^2.

Let $X\in B_{0}$ and $x\in B_{t}$ be the position vectors of material points in the initial and current configurations, respectively. These are related by the one-to-one map (we do not list time as a variable for conciseness):

(1) x=φ(X)

The associated deformation gradient mapping line elements from the initial to the current configuration is defined as^1^:

(2) F=Gradφ(X)

The bulk jacobian of the transformation maps volume elements from the initial to the current configuration: dv=JdV, with J=detF>0. We also define $f=F^{-1}$.

We define the (non-invertible) interface deformation gradient mapping line elements on the interface from the initial to the current configuration as:

(3) F^=Grad^φ(X)=Gradφ(X)⋅I^

where I^ is the surface projection tensor:

(4) I^=I−N⊗N

where I is the unit tensor in the reference configuration. The surface jacobian of the transformation maps surface elements from the initial to the current configuration: da=J^dA, with J^=det^F^=J|F−T⋅N|. The latter relation is the scalar version of the Nanson formula:

(5) $$nda=JF^{-T}\cdot NdA$$

where the surface normal in the current configuration can be related to the surface normal in the reference configuration as:

(6) $$n=\frac{N\cdot f}{\left|N\cdot f\right|}$$

We also introduce the surface projection operator in the current configuration:

(7) i^=i−n⊗n

where i is the unit tensor in the current configuration.

We describe the orientation of each liquid crystal molecule by a unit vector m (see Fig. 1), which is aligned with the major axis of the molecule. For nematic liquid crystals, the average orientation of the molecules and their degree of alignment in the liquid crystal phase can be simultaneously described using a second order, traceless, symmetric tensor Q, defined as (de Gennes and Prost, 1993):

(8) $$Q=\frac{1}{N}\underset{i}{\sum }\left(m^{\left(i\right)}⊗m^{\left(i\right)}-\frac{I}{3}\right)$$

where the summation is performed over N molecules in a representative volume element of the liquid crystal phase. Note that this expression can be applied to other types of liquid crystals to describe their orientational ordering, although additional order parameters may be required (e.g. translational order parameter for smectic liquid crystals (de Gennes and Prost, 1993)). Let $\alpha_{i}$ (i=1,2,3) be the principal values of the Q-tensor, with $\alpha_{1}+\alpha_{2}+\alpha_{3}=0$, and $d_{i}$ the corresponding principal directions. Due to its traceless property, the Q-tensor can be expressed as:

(9) $$Q=S_{1}\left(d_{1}⊗d_{1}\right)+S_{2}\left(d_{2}⊗d_{2}\right)-\frac{S_{1}+S_{2}}{3}I$$

where the parameters $S_{1}$ and $S_{2}$ are related to the principal values by

(10) $$S_{1}=2\alpha_{1}+\alpha_{2},\;S_{2}=2\alpha_{2}+\alpha_{1}$$

For uniaxial nematic liquid crystals, the Q-tensor is transversely isotropic, which implies that two of its eigenvalues coincide. Without loss of generality, let $d_{1}$ be the direction of transverse isotropy, so that $\alpha_{2}=\alpha_{3}$ and $S_{2}=0$. The Q-tensor (9) simplifies as:

(11) $$Q=S\left(d⊗d-\frac{I}{3}\right)$$

where $d\equiv d_{1}$ and $S\equiv S_{1}$. When all the molecules are perfectly aligned, it is easily seen from Eq. (8) that S=1. The case where all molecules are randomly oriented corresponds to S=0. Finally, the case where all molecules are isotropically distributed in a plane perpendicular to d corresponds to $S=-\frac{1}{2}$. Thus, the parameter S describes the degree of alignment of the molecules relative to the director. In general, $-\frac{1}{2}\leq S\leq 1$ (Andrienko, 2018). The director d and scalar order parameter S together describe the average orientation and degree of disorder of the molecules at a point in the nematic liquid crystal phase.

### 2.2 Free energy function

#### Bulk energy.

The free energy in the bulk (matrix or liquid crystal phase) is additively decomposed into elastic, distortion and residual contributions:

(12) $$U\left(F,\text{grad}\;Q;X\right)=U_{e}\left(F;X\right)+U_{Q}\left(F,\text{grad}\;Q;X\right)+U_{r}\left(F;X\right)$$

where $U_{e}$ is the free energy density of elastic deformation, $U_{Q}$ is the free energy density associated with distorting the director field, and $U_{r}$ is the energy density associated with the residual stress field. The capital letter U represents the free energy per unit volume in the reference configuration. The parametric dependence in X is used to indicate that material parameters depend on the phase.

In this paper, the soft matrix is taken to be a soft elastomer or a hydrogel, which can usually be considered as incompressible. For simplicity, the incompressible neo-Hookean model is adopted:

(13) $$U_{e}\left(F;X\right)=\frac{G\left(X\right)}{2}\left[\text{tr}\left(F^{T}\cdot F\right)-3\right]$$

where G is the shear modulus, taken uniform in each phase: $G\left(X\right)=G^{r}$ when $X\in B_{0}^{r}$ with r=m,i. Following Ghosh and Lopez-Pamies (2022), the liquid crystal inclusion is treated as a hyperelastic fluid and therefore $G^{i}/G^{m}\to 0$.

The free energy arising from the spatial variation of the order parameter is given by:

(14) $$U_{Q}\left(F,\text{grad}\;Q;X\right)=Ju_{Q}\left(\text{grad}\;Q;X\right)$$

where $u_{Q}$ is Landau–de Gennes elastic energy density (energy per unit volume in the current configuration). For a uniaxial nematic liquid crystal, a free energy form with three invariant combinations of the gradient of Q is adopted (Mottram and Newton, 2014):

(15) $$u_{Q}\left(\text{grad}\;Q;X\right)=\frac{L_{1}\left(X\right)}{2}\left(Q_{ij,k}Q_{ij,k}\right)+\frac{L_{2}\left(X\right)}{2}\left(Q_{ij,j}Q_{ik,k}\right)+\frac{L_{3}\left(X\right)}{2}\left(Q_{ik,j}Q_{ij,k}\right)$$

where the index notation $Q_{ij,k}$ refer to the gradient with respect to coordinates in the current configuration. This free energy form has been constructed based on mathematical considerations of objectivity, neglecting higher-order terms. There have been studies and proofs of the general objectivity constraint of the free energy density function, which will not be presented in this paper. Interested readers can refer to Chapter 4 in Sonnet and Virga (2012) and appendices in Zhang et al. (2019). For simplicity, we assume that all three terms equally contribute to the excess free energy due to distortion, so that $L_{1}\left(X\right)=L_{2}\left(X\right)=L_{3}\left(X\right)\equiv L\left(X\right)$ (one-constant approximation). We take L(X)=L when $X\in B_{0}^{i}$, and L(X)=0 when $X\in B_{0}^{m}$.

Due to the presence of surface tension and spatial variation of the order parameter tensor Q, a residual (first Piola–Kirchhoff) stress field $P_{r}\left(X\right)$ is in general needed to achieve mechanical equilibrium in the reference configuration. The corresponding residual energy takes the form:

(16) $$U_{r}\left(F;X\right)=P_{r}\left(X\right):F$$

In the case of spherical liquid inclusions with isotropic surface tension, the residual stress field in the inclusion is of the form $P_{r}=pJF^{-T}$ and balances the isotropic surface tension, where p is a hydrostatic pressure. The corresponding residual energy per unit volume in the inclusion is simply given by pJ (Ghosh and Lopez-Pamies, 2022). This result is nothing else than the standard Young–Laplace equation, which describes balance between the pressure on the inner side of an interface and a uniformly distributed surface tension. For a liquid inclusion with radius $R_{0}$, the pressure is $p=\frac{2\gamma }{R_{0}}$ and the residual stress in the matrix vanishes. When the (liquid) inclusion is not spherical, the non-uniform curvature of the interface leads to a non-uniform surface tension, which drives the inclusion shape towards the spherical shape. Non-spherical inclusion shape cannot be balanced solely by the pressure in the inclusion and a residual stress field in the matrix is also required to equilibrate the reference configuration. Similarly, liquid crystal inclusions of arbitrary shape and anisotropic surface tension generally require residual stress fields in both the inclusion and the matrix to equilibrate the reference configuration. The main difference with the liquid inclusion case is that the residual stress field in the liquid crystal inclusion is no longer a uniformly distributed pressure, but a stress field taking into account the contribution of the Q-field. In this work, for simplicity, we will consider that liquid crystal inclusions adopt the free-droplet shape in the reference state (i.e., the shape that the liquid crystal inclusion would adopt at equilibrium in a fluidic medium), so that the inclusion residual stress balances the anisotropic surface tension, and the residual stress in the matrix vanishes. Details about the determination of the residual stress field will be presented in Section 4.2.

#### Interface energy.

The interface free energy is taken of the following form:

(17) U^(F^,Q,N)=J^u^(Q,n)

where u^ is the interface energy per unit area in the current configuration, which is taken of the form:

(18) u^(Q,n)=γ1+ω1d⋅n2+ω2S2−S022

where γ is the surface tension, $\omega_{1}$ is the anchoring strength of the director, $\omega_{2}$ is the anchoring strength of the scalar order parameter and $S_{0}$ is the preferred scalar order parameter of the liquid crystal on the surface (Fournier and Galatola, 2005). For a tangentially anchoring interfacial behaviour, the anchoring strength $\omega_{1}$ has to be positive. In Eq. (18), the first term penalises the increase in surface area, the second term promotes the alignment of the director field with the interface plane. The first two terms together constitute the classical Rapini-Papoular form of anisotropic surface energy. The third term is added to specify the preferred scalar order parameter at the interface (Fournier and Galatola, 2005). Expression (18) is commonly adopted to describe interfaces of droplets bounded by isotropic fluids (Warenghem et al., 1984) or soft polymer interfaces (Ramdane et al., 2000). The isotropic surface energy model is recovered simply by setting $\omega_{1}=\omega_{2}=0$.

Here we assumed that the isotropic part of the interfacial energy is only dependent on a constant surface tension. This is based on the approximation that molecules at the interface are free to rearrange. This approximation is commonly adopted to describe the interface between an ideal polymer gel and a solvent, where the bulk of the surface is liquid-like (Henann and Bertoldi, 2014, Style et al., 2017). A more general treatment of surface elasticity could account for strain-dependent interfacial behaviour, which has been shown to play a role in certain circumstances (Javili and Steinmann, 2010, Heyden et al., 2021, Heyden et al., 2022). In our framework, this could be achieved by taking $\overset{ˆ}{u}$ as a function of F^.

### 2.3 Variational formulation

We obtain the governing equations for liquid crystal inclusion reinforced composites starting from a variational formulation of the boundary value problem. We consider a body subjected to a field of body force B in $B_{0}$ and to a field of external tractions T applied on its external boundary $\partial B_{0}$. The total potential energy of the body is given by:

(19) I(φ,Q,gradQ,p)=∫B0U(F,gradQ;X)dV+∫S0U^(F,Q;X,N)dS+∫B0p(J−1)dV−∫B0B⋅φdV−∫∂B0T⋅φdS

where p is a scalar Lagrange multiplier field enforcing the incompressibility constraint J=1. On the right-hand side of the above equation, the first term represents the total energy of the matrix and the inclusion, the second term is the total surface energy of the interface, the third term is the penalty term associated with incompressibility, and the last two terms represent the potential energy of the body force and external tractions, respectively. We seek to find the stationary point of the total potential energy with respect to all admissible fields φ, Q and p.

#### 2.3.1 Variation δφ

The following derivations are based on similar derivations obtained by Steinmann (2008) and Javili and Steinmann (2010) for bulk elastic and surface energies, now including the orientation energy of liquid crystal following Virga (2019). We consider the first variation from φ→φ+ɛδφ around the true solution (φ,Q,p). The corresponding variation of the potential energy is identified as:

(20) $$\delta_{\varphi }I=\frac{d}{dɛ}\left[I\left(\varphi +ɛ\delta \varphi ,Q,p\right)\right]{|}_{ɛ=0}$$

The true solution corresponds to a stationary point of the functional: $\delta_{\varphi }I=0$, solving which gives us the following weak form:

(21) ∫B0P:GradδφdV+∫S0P^:Grad^δφdS=∫B0B⋅δφdV+∫∂B0T⋅δφdS

where P represents the bulk first Piola–Kirchhoff stress, here given by:

(22) $$P=P_{e}+pJf^{T}+Ju_{Q}f^{T}+J\sigma^{Q}\cdot f^{T}+P_{r}$$

where $P_{e}$ is the stress derived from the elastic strain energy (13) and given by:

(23) $$P_{e}=\frac{\partial U_{e}}{\partial F}=G\left(X\right)F$$

In Eq. (22), p physically represents the hydrostatic pressure arising from the incompressibility constraint. The term $Ju_{Q}f^{T}$ represents the Landau–de Gennes energy in the reference configuration under given deformation gradient. $\sigma^{Q}$ arises from the distortion of the Q-field, which is analogous to the Ericksen stress (Ericksen, 1962) and is given by:

(24) $$\sigma^{Q}=-\text{grad}\;Q⊙\frac{\partial u_{Q}}{\partial \text{grad}\;Q}$$

or, in index notation:

(25) $$\sigma_{ij}^{Q}=-Q_{kl,i}\frac{\partial u_{Q}}{\partial Q_{kl,j}}$$

With $u_{Q}$ of the form (15) under the one-constant approximation, the latter expression becomes:

(26) $$\sigma_{ij}^{Q}=-L\left(X\right)Q_{kl,i}\left(Q_{kl,j}+\delta_{lj}Q_{km,m}+Q_{kj,l}\right)$$

In Eq. (21), P^ represents the surface first Piola–Kirchhoff stress and can be decomposed as:

(27) P^=J^u^f^T+n⊗S^0

where S^0 is the so-called deformational surface shear (Steinmann, 2008):

(28) S^0=π^t⋅J^f^Twithπ^t=−∂u^∂n⋅i^

Inserting Eq. (17) into Eq. (28), we obtain:

(29) π^t=−2γω1cos〈d,n〉(d−cos〈d,n〉n)

S^0=−2J^γω1cos〈d,n〉(d−cos〈d,n〉n)f^T

where 〈d,n〉 represents the angle between the director and the surface normal in the current configuration. The first term in Eq. (27) describes the contribution to the surface stress due to increasing the surface area, and the second term represents the contribution to the surface stress due to anchoring effects. In the particular case where $\omega_{1}=\omega_{2}=0$, u^=γ, and the surface stress reduces to P^=J^γf^T.

We illustrate the physical interpretation of surface shear in a simple 2D example in 2D. In a 2D Cartesian coordinate system with basis vectors $\left\{e_{x},e_{y}\right\}$, the director field can be written as: $d=\cos\theta e_{x}+\sin\theta e_{y}$, where θ represents the angle between the director and $e_{x}$. The liquid crystal is in contact with a flat surface with normal vector $n=e_{y}$. In this case, the deformational surface shear in the current configuration is obtained as:

(30) π^t=−γω1sin2θex

From this expression we can easily see that when θ=kπ/2, k=0,±1,±2,…, π^t=0, which corresponds to cases where the director is either parallel or perpendicular to the surface. When θ=π/4+kπ/2, the surface shear reaches its maximum absolute value. Thus, there exists a tendency for the director field to be reoriented towards the surface tangential plane, or equivalently, for the surface to be tilted along the director. The resultant droplet shape and director field are dependent on the relative strength between the surface and distortional energy.

Using integration by part and the divergence theorem in the bulk and on the interface, the following strong form can be obtained from Eq. (21):

(31) $$\text{Div}\;P+B=0\;\text{in}\;B_{0}$$

(32) $$P\cdot N=T\;\text{on}\;\partial B_{0}$$

(33) Div^P^=(Pi−Pm)⋅NonS0

(34) P^⋅N^1+P^⋅N^2=0onC0

Eqs. (31)–(34) recover the classical mechanics equations in the presence of surface stresses (Steinmann, 2008). The only difference is the Landau–de Gennes elasticity contribution included in the expression (22) of the total stress. In particular, Eq. (34) represents the condition of force balance at the junction curve $C_{0}$. This condition has been used to enforce mechanical equilibrium at the interface between three or more phases (Simha and Bhattacharya, 2000, Henann and Bertoldi, 2014). In the context of liquid crystal problems, such junction curve has been theoretically shown to occur when the anchoring strength $\omega_{1}$ is negative (where directors prefer to be anchored perpendicular to the interface) (Virga, 2019). In the rest of the paper, we only consider cases where the anchoring strength is positive and the inclusion surface does not have any junction curve. We refer to Appendix A.1 for derivations details of the weak and strong form.

#### 2.3.2 Variation δQ

Now we look at the first variation from Q→Q+ηδQ around the true solution:

(35) $$\delta_{Q}I=\frac{d}{d\eta }\left[I\left(\varphi ,Q+\eta \delta Q,p\right)\right]{|}_{\eta =0}$$

Solving $\delta_{Q}I=0$, we arrive at the weak form:

(36) ∫BtsQ⋮gradδQdV+∫St∂u^∂Q:δQdS=0

where $s^{Q}$ is the torque stress, given by:

(37) $$s^{Q}=\frac{\partial u_{Q}}{\partial \text{grad}\;Q}$$

The torque stress can be interpreted as an analogue to the elastic stress, except that it conjugates with the gradient of the order parameter Q. Using Eq. (15), the torque stress specialises as:

(38) $$s_{ij,k}^{Q}=Q_{ij,k}+\delta_{jk}Q_{im,m}+Q_{ik,j}$$

In Eq. (36), the operator ⋮ is defined as:

(39) $$A_{ijk}e_{i}⊗e_{j}⊗e_{k}\;\vdots \;B_{lmn}e_{l}⊗e_{m}⊗e_{n}=A_{ijk}B_{ijk}$$

Using again integration by parts and the divergence theorem, the corresponding strong form of the governing equations for the order parameter are obtained:

(40) $$\text{div}s^{Q}=0\;\text{in}\;B_{t}$$

(41) sQ⋅n+∂u^∂Q=0onSt

(42) $$s^{Q}\cdot n=0\;\text{on}\;\partial B_{t}$$

Eqs. (40)–(42) recover the governing equations for liquid crystal problems presented in Sonnet and Virga (2012).

#### 2.3.3 Variation δp

Finally, we take the first variation from p→p+ξδp, which gives the following condition:

(43) $$\int_{B_{0}}\left(J-1\right)\delta p\;dV=0$$

The corresponding strong form is equivalent to the incompressibility constraint J=1.

### 2.4 Summary of the governing equations

In summary, the solution of the boundary-value problem for a liquid crystal inclusion in an elastic matrix subjected to a field of mechanical body forces and surface traction can be stated as follows.

#### Weak form.

Find (φ,Q,p) that satisfy:

(44) ∫B0P:GradδφdV+∫S0P^:Grad^δφdS−∫B0B⋅δφdV−∫∂B0T⋅δφdS=0

(45) ∫B0JsQ⋮gradδQdV+∫S0J^∂u^∂Q:δQdS=0

(46) $$\int_{B_{0}}\left(J-1\right)\delta p\;dV=0$$

together with the constitutive relations for P, P^, $s^{Q}$ and $\overset{ˆ}{u}$.

#### Strong form.

Find (φ,Q,p) that satisfy:

(47) $$\text{Div}\;P+B=0\;\text{in}\;B_{0}$$

(48) $$\text{div}\;s^{Q}=0\;\text{in}\;B_{t}$$

(49) $$J=1\;\text{in}\;B_{0}$$

together with the following boundary and interface conditions:

(50) $$P\cdot N=T\;\text{on}\;\partial B_{0}$$

(51) Div^P^=(Pi−Pm)⋅NonS0

(52) sQ⋅n+∂u^∂Q=0onSt

(53) P^⋅N^1+P^⋅N^2=0onC0

and the constitutive relations.

## 3 Numerical implementation

We have implemented the theory into a finite element code using the open source finite element library FEniCSx (Alnaes et al., 2015). The code is freely available on Zenodo (Bai and Brassart, 2025).

### 3.1 Geometry and mesh

**Fig. 3:**
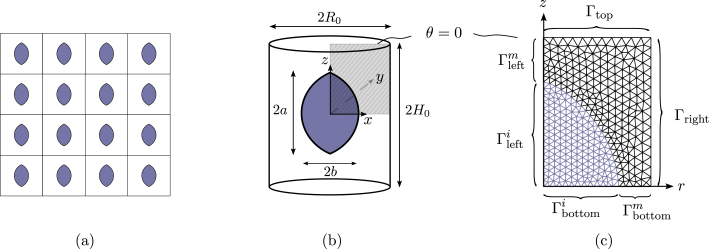
(a) Schematic of the periodic composite; (b) Approximation using axisymmetric unit cell; (c) 2D finite element mesh.

We consider a periodic composite consisting of aligned axisymmetric prolate liquid crystal inclusions arranged on a rectangular lattice (Fig. 3). The assumption of axisymmetric inclusions is motivated by experimental observations reported by Kaznacheev et al. (2002), which show that the shape of the liquid crystal droplet and the director field are axisymmetric in the natural state. We also assume that the interface consists of a single smooth surface, i.e. we do not account for line interfaces. Virga (2019) (Chapter 5) has shown that when the interfacial energy is represented by the Rapini-Papoular model, there is no line singularity on the interface when the anchoring effect is tangential to the interface (i.e. $\omega_{1}>0$). We write ϕ the volume fraction of the inclusions. The inclusions have semi-major axis a in the z-direction, and semi-minor axis b in the x- and y-directions. The aspect ratio of the inclusions is defined as $\epsilon =\frac{a}{b}\geq 1$. For simplicity, and to reduce the computational cost, we represent the composite using an axisymmetric Unit Cell (UC) with radius $R_{0}$ and height $2H_{0}$ and containing a single inclusion at its centre (Fig. 3b). We consider the aspect ratio of the UC to be identical to the inclusion aspect ratio: $\frac{H_{0}}{R_{0}}=\epsilon$. The volume fraction of the inclusion is given by $\phi =\frac{V^{i}}{V}$ where $V^{i}$ is the inclusion volume and $V=2\pi H_{0}R_{0}^{2}$ is the UC volume. The geometry is defined in a cylindrical coordinate system with origin at the centre of the inclusion and fixed polar angle θ=0, and with basis vector $\left\{e_{r},e_{z}\right\}$. The geometry is discretised in a 2D triangular mesh using the software GMSH (Geuzaine and Remacle, 2009), accounting for axisymmetry and periodicity, as shown in Fig. 3c. The simulations in this paper use about 400 elements and we have verified that the number of elements was sufficient to achieve convergence.

The numerical solution obtained using this 2D axisymmetric UC is an approximation of the true solution for the composite shown in Fig. 3a, and is limited to the case where the loading conditions are symmetric with respect to the major axis. We adopted the axisymmetric approximation because it is sufficient to investigate the main coupling effects in these composites, while keeping the computational cost low. For comparison, we have also implemented our theory in 3D. We have verified that the 2D axisymmetric approximation is in good agreement with reference calculations obtained on 3D cells, see Appendix C.

### 3.2 Mixed finite element formulation

As stated in Section 2, the variables of the boundary value problem consist of φ,Q and p, which are vector, tensor and scalar, respectively. We adopt a standard mixed formulation for the displacement field and pressure field, see Chapter 8 in Boffi et al. (2013), that is, second order continuous Galerkin elements (CG-2) for the displacement field and zeroth order (scalar) discrete Galerkin element (DG-0) for the pressure field, which is proved to be stable for triangular elements. As for the Q-field, first note that in the 2D axisymmetric case, the Q-tensor only has two independent components: the scalar order parameter S and one independent component of the director d (the other two components are constrained by the condition |d|=1 and axisymmetry). We parametrise d using the angle ψ between the director and the r-axis so that $d=\cos\psi e_{r}+\sin\psi e_{z}$. The Q-tensor is then parametrised in terms of ψ and the scalar order parameter S:

(54) $$\left[Q\right]=S\left[\begin{array}{ccc} \cos^{2}\psi -\frac{1}{3} & 0 & \cos\psi \sin\psi \\ 0 & -\frac{1}{3} & 0\\ \cos\psi \sin\psi & 0 & \sin^{2}\psi -\frac{1}{3} \end{array}\right]$$

We interpolate the scalar functions ψ and S with first-order continuous Galerkin elements (CG-1). When treating the problem with axisymmetric elements, we also need expressions of the gradient of vector and tensor fields in cylindrical coordinates. Expressions for these operators can be found in Appendix B. In addition, since state variables ψ and S are only defined in the inclusion domain, $B_{0}^{i}$, we used the package multiphenicsx (Ballarin, 2024) to restrict the variables within the inclusion subdomain.

### 3.3 Dimensionless parameters

In our parametric study, we focus mainly on the effect of three dimensionless material parameters: the relative anchoring strength $\omega_{1}$, the dimensionless inclusion volume v and the elasto-capillary number ξ. The physical meaning of these parameters is described below.

The relative anchoring strength $\omega_{1}$ quantifies the relative contributions of surface anchoring and surface tension, as evident from Eq. (18).

The dimensionless volume v characterises the tendency of the director field in an inclusion of a given size to be distorted as a result of interface anchoring. The ratio of distortional elastic constant L (energy per length) to the anchoring energy $\gamma \omega_{1}$ (energy per area) defines a material length, known as the surface extrapolation length (de Gennes and Prost, 1993):

(55) $$\ell =\frac{L}{\gamma \omega_{1}}$$

The surface extrapolation length represents the distance from the actual interface to an imaginary surface where the director is completely aligned with the surface. In other words, ℓ sets the length scale for the distortion of the director field due to an anchoring surface. When ℓ is “large”, distortional elasticity prevails over surface anchoring. The extrapolation length should be compared to the characteristic size of the inclusion. To this end, the dimensionless inclusion volume (Prinsen and van der Schoot, 2003) is introduced:

(56) $$v=\frac{V^{i}}{\ell^{3}}$$

When the inclusion size is small relative to the extrapolation length (v≪1), the director field is unable to reorient over the inclusion length scale and remains relatively unaffected by surface anchoring. When the inclusion size is large relative to the extrapolation length (v≫1), the director is able to reorient and to align with the interface. The ability of the director field to anchor at the interface thus depends on the inclusion size.

The elasto-capillary number ξ is defined as:

(57) $$\xi =\frac{\gamma }{G^{m}\overset{\sim }{R}}$$

where $\overset{\sim }{R}$ is a characteristic size of the inclusion related to the inclusion volume as:

(58) $$V^{i}=\frac{4}{3}\pi {\overset{\sim }{R}}^{3}$$

The elasto-capillary number describes the relative strength of surface tension relative to the elasticity of the matrix. When the elasto-capillary number is small (ξ→0), the matrix deformation is unaffected by the surface tension at the interface. Elasto-capillary effects become significant (ξ>1) when the inclusion is small and/or the matrix is soft.

Additional dimensionless parameters are $\omega_{2}$ and $S_{0}$, which dictate the evolution of the order parameter S. However, the order parameter does not affect the orientation of the director field, which is our primary interest in this study. Therefore, in this paper, we adopt the value of $S_{0}=1$ and set $\omega_{2}=20$ in all our simulations.

### 3.4 Bipolarness

**Fig. 4:**
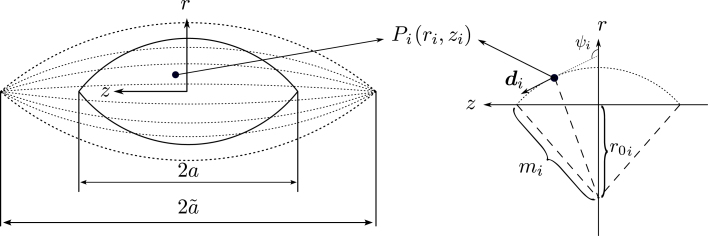
Approximation of the director field inside the inclusion using circular arcs. The ratio $\frac{a}{\overset{\sim }{a}}$ defines the bipolarness of the director field.

We adopt the concept of “bipolarness” introduced by Prinsen and van der Schoot (2004) to describe the director field within a liquid crystal droplet. In this representation, the directors are assumed to be oriented tangentially to revolving circular arcs with equation:

(59) $${\left(r+r_{0}\right)}^{2}+z^{2}=m^{2}$$

where m is the radius of the circle and $r_{0}>0$ is the distance between the origin and the centre of the circle, see Fig. 4. The intersection of the circular arcs with the z-axis is denoted $\overset{\sim }{a}$. The ratio between $\overset{\sim }{a}$ and a can then be used as a measure of the degree of distortion of the director field inside a droplet. When $\frac{a}{\overset{\sim }{a}}\to 1$, the circular arcs representing the director orientations meet the tips of the droplet, and the director field inside the droplet is perfectly bipolar. When $\frac{a}{\overset{\sim }{a}}\to 0$, the circular arcs are parallel straight lines, corresponding to complete alignment of the directors inside the droplet.

In practice, we obtain the equation of the circular arcs from finite element simulation results in the following way. For a given node $P_{i}$ with coordinates $\left(r_{i},z_{i}\right)$ and director orientation $\psi_{i}$, Eq. (59) particularises as:

(60) $${\left(r_{i}+{r_{0}}_{i}\right)}^{2}+z_{i}^{2}=m_{i}^{2}$$

By definition, the director is tangential to the arc at this point, giving:

(61) $$\sin\left(\psi_{i}-\frac{\pi }{2}\right)=\frac{z_{i}}{m_{i}}$$

As shown in the next section, for x>0 and z>0, ψ is always larger than $\frac{\pi }{2}$. Eq. (61) gives the radius of the circle $m_{i}$ at node $P_{i}$. Substituting into Eq. (60) gives the distance ${r_{0}}_{i}$ as:

(62) $${r_{0}}_{i}=-r_{i}+\frac{z_{i}}{\tan\left(\psi_{i}-\frac{\pi }{2}\right)}$$

The distance between the tip of the circular arc and the origin is obtained as:

(63) $${\overset{\sim }{a}}_{i}=\sqrt{m_{i}^{2}-{r_{0}}_{i}^{2}}$$

Since the representation of the director field orientation using circular arcs is not exact, the distance ${\overset{\sim }{a}}_{i}$ varies from node to node. Therefore, we consider the average bipolarness of the director field:

(64) $$\frac{a}{\overset{¯}{\overset{\sim }{a}}}=\frac{a}{N}\overset{N}{\underset{i}{\sum }}\frac{1}{{\overset{\sim }{a}}_{i}}$$

where N is the number of nodes in the inclusion domain.

## 4 Results

In this section, we consider two classes of problems to illustrate the underlying physics of the composite system through parametric analyses. The first one (Section 4.1) is the free droplet problem, where we investigate the equilibrium shape and director field of a liquid crystal droplet in an isotropic fluid environment. The second one consists of the problem of an inclusion embedded in a soft matrix and subjected to external mechanical loads.

### 4.1 Free droplet

#### Simulation method.

When the inclusion is surrounded by a fluidic environment, the problem reduces to the classical problem of finding the equilibrium shape of a liquid crystal droplet with deformable boundaries (Kaznacheev et al., 2002, Prinsen and van der Schoot, 2003, Prinsen and van der Schoot, 2004). The equilibrium shape of the droplet is then dictated by the competition between the interfacial energy and the distortional energy of the liquid crystal. Previously, the numerical modelling on the free droplet problem has been investigated through Monte Carlo simulations (Bates, 2003), molecular dynamics (Vanzo et al., 2012), phase field simulations (Diegel and Walker, 2017) as well as staggered algorithms (DeBenedictis and Atherton, 2016, Adler et al., 2023), i.e. iteratively solving for the order parameter field with a fixed interface, and for the inclusion shape with fixed order parameter. In contrast, in our approach we obtain the Q-tensor field and the shape of the inclusion simultaneously for a finite value of the shear modulus $G^{m}=G^{i}$, and iteratively reduce the moduli until reaching the equilibrium shape, as explained below.

For the reference configuration, we consider a spherical droplet with radius $\overset{\sim }{R}$ embedded in a matrix. In the reference configuration, both the matrix and the inclusion are endowed with bulk elastic energy of the form (13), with $G^{m}=G^{i}=G^{\text{initial}}$, and we do not consider surface tension. In this case, the reference configuration is at equilibrium without any residual stress inside the droplet, and the order parameter is uniform in the inclusion. The boundary conditions are:

(65) $$\varphi_{r}=0\;\text{on}\;\Gamma_{\text{left}}$$

$$\varphi_{z}=0\;\text{on}\;\Gamma_{\text{bottom}}$$

$$\sigma \cdot n=0\;\text{on}\;\Gamma_{\text{right}}\cup \Gamma_{\text{top}}$$

$$\;\psi =\frac{\pi }{2},\;\frac{\partial S}{\partial n}=0\;\;\text{on}\;\Gamma_{\text{left}}^{i}\cup \Gamma_{\text{bottom}}^{i}$$

Here, we used Dirichlet boundary conditions for the angle ψ on $\Gamma_{\text{left}}^{i}\cup \Gamma_{\text{bottom}}^{i}$, instead of the natural boundary condition $\frac{\partial \psi }{\partial n}=0$. We have verified that the numerical solution obtained using natural boundary conditions indeed gives ψ=π/2+kπ, k=0,±1,±2,… (the other admissible solution in terms of symmetry would be ψ=0+kπ). We adopted the Dirichlet boundary condition in the parametric study because it facilitated convergence and avoids the multiple solutions of ψ due to periodicity. We then apply the (anisotropic) surface tension, similar to an external load (Henann and Bertoldi, 2014). The applied surface tension is ramped gradually to facilitate convergence. Activating the anisotropic surface tension tends to deform the droplet shape and in turn distorts the order parameter field, while the droplet deformation is resisted by the elastic matrix. After having equilibrated the system, we then decrease the shear moduli of both the matrix and the inclusion, and recalculate the new equilibrium shape and director field. The procedure is repeated for gradually decreasing shear moduli, $G^{m}=G^{i}$, until the droplet reaches its fully relaxed configuration, where anisotropic surface tension exactly balances the tendency of the directors to align. The relaxed configuration is then independent of the actual value of the shear moduli $G^{m}$ and $G^{i}$. In practice, we consider that the droplet is fully relaxed when the change in its aspect ratio △ϵ between two increments in shear moduli is smaller than 10^−4^. Note that during relaxation, the mesh can be distorted significantly, causing convergence issues before the tolerance is reached. In such cases, we re-mesh the domain whenever the solver fails to converge and continue the relaxation process with the new mesh. We verified that the equilibrium shape of the droplet is independent on the volume fraction of the inclusion, as expected.

#### Comparison to experimental data.

**Fig. 5:**
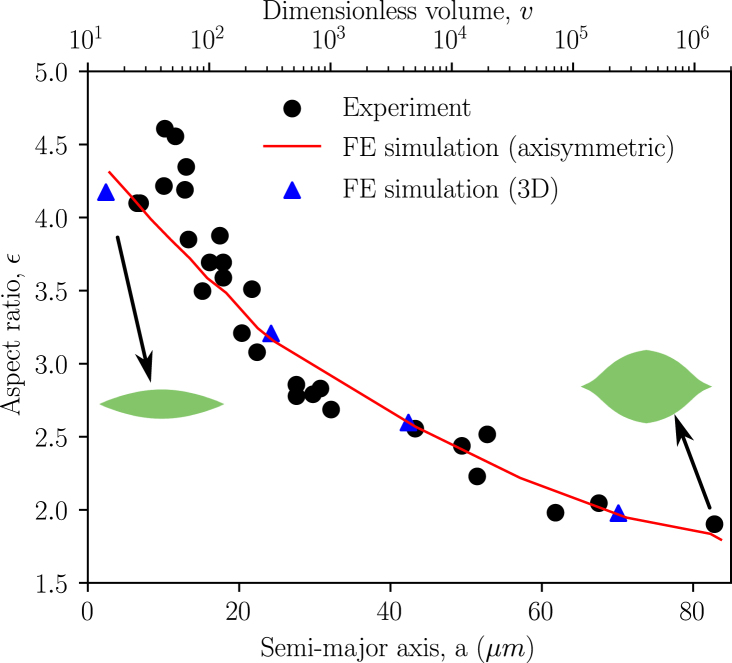
Comparison between experimental and finite element simulation results for the evolution of the droplet shape (aspect ratio, ϵ) with the inclusion size (semi-major axis a, or dimensionless volume v). Parameters were identified as: $\omega_{1}=5$, $\gamma =0.003\;\text{J}/{\text{m}}^{2}$, and $L=10^{-8}\;\text{J}/\text{m}$.

To validate our model, we compare numerical predictions to experimental data reported by Kaznacheev et al. (2002) for a dispersion of 0.33 wt% of vanadium pentoxide in water. In that study, snapshots of equilibrated droplets in solution were taken, and the aspect ratio and semi-major axis of each droplet were measured. Material parameters were set as follows: $\omega_{1}=5$, γ=0.003 J/m${}^{2}$ and $L=10^{-8}$ J/m, which were fitted to experimental data. Liquid crystal droplets with varying volume $V^{i}$ (i.e. different characteristic sizes $\overset{\sim }{R}$) were simulated. For each $\overset{\sim }{R}$, we calculated the equilibrium semi-major axis a and aspect ratio $\epsilon =\frac{a}{b}$. The comparison between simulation results and experimental data is shown in Fig. 5, showing good agreement. In addition to 2D simulations using axisymmetric unit cells, we also carried out 3D simulations. Numerical details of 3D simulations are provided in Appendix C. As expected, simulations results in 2D and 3D are in very good agreement, since the axisymmetric approximation is exact for the free droplet problem. Results show that, when the volume of the inclusion increases (i.e. the dimensionless volume v increases), the aspect ratio decreases. Indeed, increasing the dimensionless volume facilitates the disorientation of the director field, allowing the droplet to adopt a more circular shape to minimise its surface energy. In contrast, at small dimensionless volume, the director field remains strongly aligned, forcing the droplet to adopt a more elongated shape to reduce the cost of interfacial misalignment.

#### Parametric study.

**Fig. 6:**
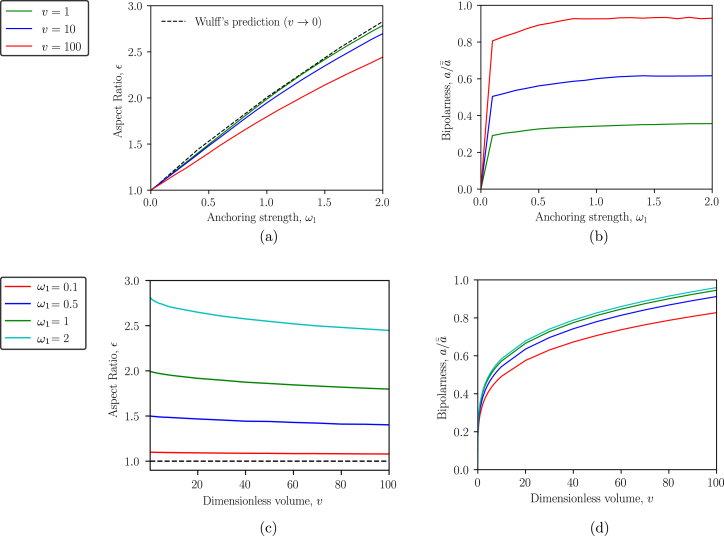
Predicted evolutions of the aspect ratio ϵ and bipolarness $\frac{a}{\overset{¯}{\overset{\sim }{a}}}$ as a function of (a)–(b) the anchoring strength $\omega_{1}$ and (c)–(d) the dimensionless volume v. Other parameters were set as $L=10^{-8}$ J/m and γ=0.003 J/${\text{m}}^{2}$.

We conducted a parametric study to assess the effect of the relative anchoring strength $\omega_{1}$ for different values of v, adjusted via the inclusion size $\overset{\sim }{R}$ at constant $L=10^{-8}$ J/m and γ=0.003 J/${\text{m}}^{2}$. The evolution of the droplet aspect ratio is shown as a function of the relative anchoring strength $\omega_{1}$ in Fig. 6a for three values of v, and the corresponding evolution of the bipolarness is shown in Fig. 6b. The figure also shows the estimate obtained using the Wulff’s construction method (Virga, 2019), according to which the equilibrium shape of an incompressible body is obtained by minimising a known functional. Wulff’s method applies only in the limit v→0, corresponding to a perfectly ordered liquid crystal. In the case where $\omega_{1}=0$, there is no cost associated with misaligning the director with the surface and the droplet adopts a spherical shape (ϵ=1). Inside the droplet, the director is perfectly aligned to minimise the distortional energy and the bipolarness tends to zero. As $\omega_{1}$ increases, due to anisotropic surface tension, the aspect ratio of the droplet increases and the bipolarness also increases.

The change of aspect ratio with an increasing dimensionless volume is shown in Fig. 6c. For a given anchoring strength, the aspect ratio decreases as the dimensionless volume increases, and tends to one for v→∞. We also show the evolution of the bipolarness of the director field in Fig. 6d. The bipolarness tends to zero as v→0, because the cost of misaligning the director field becomes much larger than the cost of misalignment at the interface, and the director field becomes nearly uniform. As v increases, the director field becomes able to reorient to align with the surface, and the bipolarness increases.

**Fig. 7:**
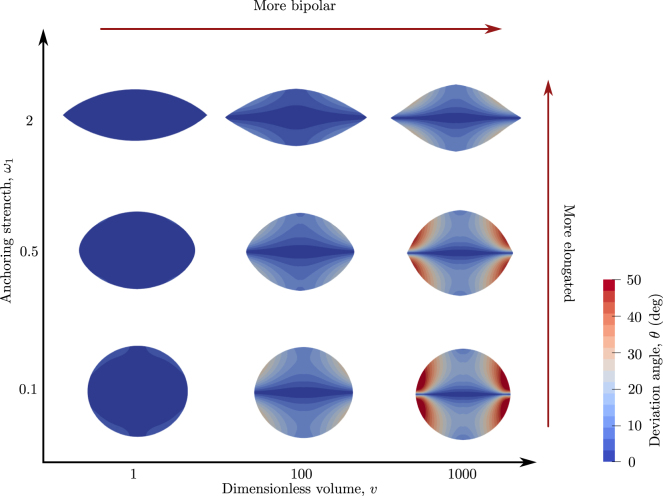
Illustration of inclusion shape and director field orientation in liquid crystal droplets with different anchoring strength and dimensionless volume.

Fig. 7 illustrates the shape of the droplet and orientation of the director field for different combinations of $\omega_{1}$ and v. The contour plots represent the deviation angle $\theta =\left(\psi -\frac{\pi }{2}\right)$ between the director and the z-axis. Note that the deviation angle was only calculated in the meshed region x≥0, z≥0, where ψ is larger than $\frac{\psi }{2}$, and mirrored in the three other quadrants for visualisation. When the dimensionless volume is small (v=1), the director field is almost uniaxial and aligned with the symmetry axis, and the deviation angle is close to zero. Increasing the anchoring strength elongates the droplet, with the appearance of point defects (called “boojums”) visible for $\omega_{1}=2$. The droplet is then referred to as “tactoid”. As the dimensionless volume increases, the local deviation angle becomes heterogeneous to reduce misalignment with the interface. At large v and small $\omega_{1}$, the droplet is able to preserve a roughly spherical shape by allowing large distortions of the director field near the surface. At large v and large $\omega_{1}$, the tactoid shape becomes more complex as a result of the competition between distortional energy and anisotropic surface energy.

### 4.2 Liquid crystal inclusion in a soft matrix

#### Reference configuration.

We hypothesise that inclusions in the composite initially have the same shape and director field orientation as the corresponding inclusions in a liquid. By adopting the shape and director field of the fully-relaxed free droplet in the reference configuration of the composite problem, we ensure that the matrix in the reference configuration is stress-free. Let $Q^{\text{FD}}$ be the order parameter and $p^{\text{FD}}$ the hydrostatic pressure in the relaxed free droplet. According to Eq. (22), the residual stress $P_{r}$ that balances the surface tension and the distortion of the order parameter is given by:

(66) $$P_{r}^{i}=u^{Q}\left(\text{grad}\;Q^{FD}\right)JF^{-T}+J\sigma^{Q}\left(\text{grad}\;Q^{FD}\right)F^{-T}+p^{FD}JF^{-T},\;P_{r}^{m}=0$$

In practice, we first solve the free droplet problem using the methodology described in the previous section, and obtain the inclusion shape and the Q-field. We then calculate the residual stress field from Eq. (66). Next, we create a new mesh on the free droplet geometry and interpolate the numerical values of $P_{r}^{i}$ onto the new mesh. The inclusion is then embedded in the soft matrix, and the matrix domain is also meshed. The self-equilibrated free-droplet embedded in the matrix is used as the reference configuration (with F=I) for subsequent composite simulations. Note that as the interpolation is performed on two different meshes, it leads to a numerical error. However, we have verified that the numerical error on the stress field is small so that it does not impact subsequent results.

#### Boundary conditions.

We subject the composite UC to uniaxial tension and compression tests in the z-direction, which satisfy the condition of axisymmetry. Similar to the free droplet problem, the composite is subjected to symmetric boundary conditions on $\Gamma_{\text{left}}=\Gamma_{\text{left}}^{i}\cup \Gamma_{\text{left}}^{m}$ and $\Gamma_{\text{bottom}}=\Gamma_{\text{bottom}}^{i}\cup \Gamma_{\text{bottom}}^{m}$:

(67) $$\varphi_{x}=0\;\text{on}\;\Gamma_{\text{left}}$$

$$\varphi_{z}=0\;\text{on}\;\Gamma_{\text{bottom}}$$

$$\psi =\frac{\pi }{2},\;\frac{\partial S}{\partial n}=0\;\;\text{on}\;\Gamma_{\text{left}}^{i}\cup \Gamma_{\text{bottom}}^{i}$$

We apply prescribed displacement boundary conditions on the top edge:

(68) $$\varphi_{z}=\left(\lambda_{z}-1\right)H_{0}\;\text{on}\;\Gamma_{\text{top}}$$

where $\lambda_{z}$ is the stretch in z-direction. The right edge is kept straight to account for periodicity, and hence interactions between neighbouring inclusions (subject to the approximation of axisymmetric UC). This constraint is weakly imposed to the edge by adding the following term to the total potential:

(69) $$\int_{\Gamma_{\text{right}}}\beta \left(\frac{\partial \varphi_{r}}{\partial z}\right)\;dS$$

where β is a Lagrange multiplier defined only on the right edge using the multiphenicsx package (Ballarin, 2024).

In the following, we conduct a parametric study to explore the role of various parameters on the macroscopic response of the composite. We also investigate the effect of mechanical loads on the orientation of the director field. In the simulations below, the Landau–de Gennes elastic constant is $L=10^{-8}\text{J}/\text{m}$, and the surface tension is $\gamma =0.003\text{J}/{\text{m}}^{2}$. We vary the value of v by changing the value of $\overset{\sim }{R}$. We change the value of ξ by changing the value of $G^{m}$. The shear modulus of the inclusion is selected as: $G^{i}/G^{m}=10^{-4}$. We have conducted a convergence study to verify that this ratio is sufficiently small. The volume fraction of the liquid crystal inclusion is kept constant: ϕ=0.2.

#### Parametric study.

**Fig. 8:**
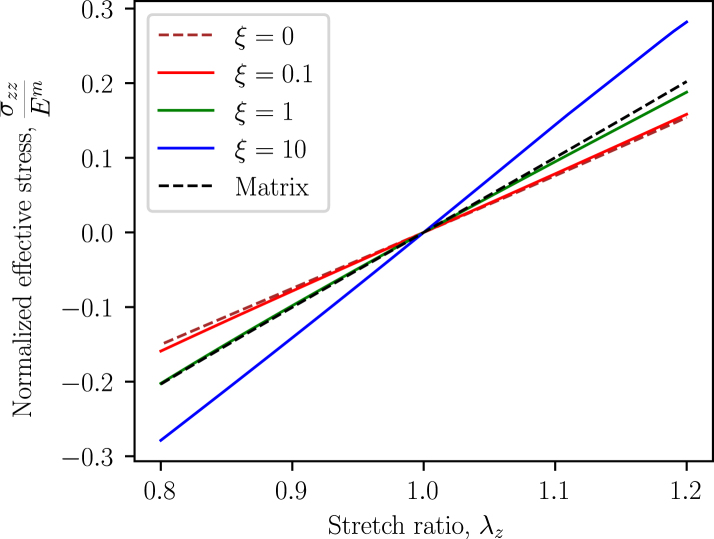
Normalised stress–stretch curves for composites made of liquid crystal inclusions embedded in an elastic matrix under uniaxial tension and compression loading. Material parameters are: $v=100,\omega_{1}=0.5$.

We first consider the stress–strain response of the composite subjected to uniaxial tension and compression tests. Fig. 8 shows the effective Cauchy stress ${\overset{¯}{\sigma }}_{zz}$ (calculated by dividing the resultant force on the top surface by the current area) normalised by the Young modulus of the matrix ($E^{m}=3G^{m}$), for different values of ξ. The anchoring strength and the dimensionless volume are $\omega_{1}=0.5$ and v=100. For comparison, we have also plotted the response of the composite when the inclusion is replaced with an incompressible isotropic liquid and negligible surface tension (ξ=0). When ξ=0.1, the elasto-capillary effect is weak and the response of the composite is softer than that of the matrix and only slightly stiffer than that of a composite with liquid inclusion and no surface tension effects. As ξ increases by decreasing the matrix modulus, the composite modulus also decreases, but not as fast as the matrix modulus, so that for ξ=10, the composite is stiffer than the matrix. These results parallel previously-reported results for liquid inclusions in soft matrices, see Style et al., 2015a, Style et al., 2015b, Wang and Henann, 2016, Ghosh and Lopez-Pamies, 2022.

**Fig. 9:**
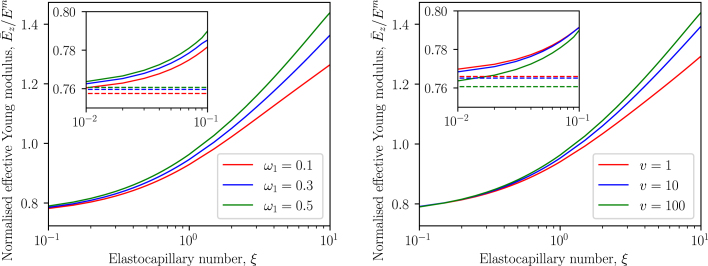
Evolution of the normalised effective Young modulus of the composite with the elasto-capillary number ξ for (a) different values of anchoring strength $\omega_{1}$ at fixed dimensionless volume v=100, and (b) different values of dimensionless volume v at fixed anchoring strength $\omega_{1}$. The insets also compare the response of the composite with liquid crystal inclusions (continuous lines) to the response of a composite containing incompressible liquid inclusions with the same shape as in the original composite and with no surface tension effects (dashed lines).

**Fig. 10:**
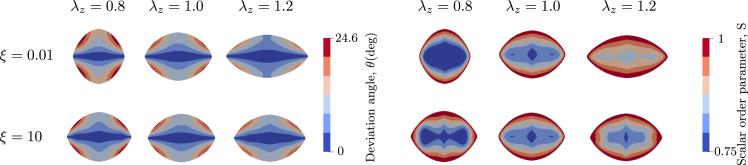
The response of liquid crystal inclusions in a soft matrix under external loading. Contour plots of the director deviation angle θ (left) and scalar order parameter S (right). Material parameters are v=100 and $\omega_{1}=0.5$.

**Fig. 11:**
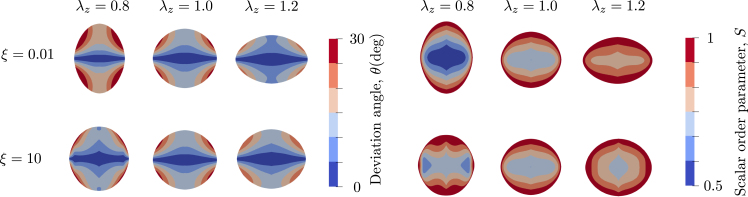
The response of liquid crystal inclusions in a soft matrix under external loading. Contour plots of the director deviation angle θ (left) and scalar order parameter S (right). Material parameters are v=100 and $\omega_{1}=0.1$.

**Fig. 12:**
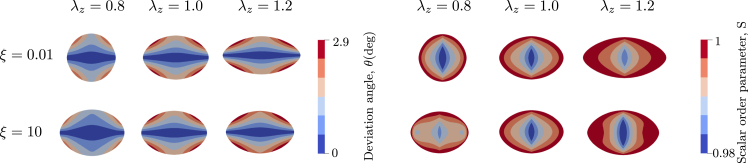
The response of liquid crystal inclusions in a soft matrix under external loading. Contour plots of the director deviation angle θ (left) and scalar order parameter S (right). Material parameters are v=1 and $\omega_{1}=0.5$.

Fig. 9 shows the effective Young modulus of the composite in the z-direction, ${\overset{¯}{E}}_{z}$, normalised by the matrix modulus as a function of the elasto-capillary number ξ, for different values of the anchoring strength and dimensionless volume. Each combination $\left(v,\omega_{1}\right)$ corresponds to a given self-equilibrated configuration of the droplet, as described in Section 4.1, while the elasto-capillary number ξ is varied by changing the matrix stiffness. As noted previously, the normalised composite modulus increases with ξ at constant values of v and $\omega_{1}$. For a fixed dimensionless volume (v=100, Fig. 9a), the effective modulus increases when the anchoring strength increases. This can be explained based on the difference in aspect ratio of the underlying free droplet: indeed, the aspect ratio increases from ϵ=1.08 for $\omega_{1}=0.1$ to ϵ=1.4 for ω=0.5 (see also Fig. 6). It is well established that the longitudinal modulus of composites reinforced by elongated inclusions increases with the inclusion aspect ratio (Lee and Paul, 2005). When ξ tends to zero, the effective modulus of the composite with liquid crystal inclusion approaches the response of a composite with the same initial inclusion shape but filled with an isotropic incompressible liquid and no surface tension, as illustrated in dashed lines in the inset of Fig. 9a. This indicates that in the small-ξ limit (reached by increasing the matrix modulus), the contributions due to anisotropic surface tension and distortional energy of the liquid crystal phase are negligible compared to the elastic energy of the matrix. We have verified that these trends remain the same at smaller values of v.

For a fixed anchoring strength ($\omega_{1}=0.5$, Fig. 9b), the effective modulus decreases when the dimensionless volume decreases at given ξ≳0.15. In this case, the aspect ratios of the inclusion in the reference configuration are very close: ϵ=1.49 for v=1 and ϵ=1.4 for v=100. However, the two inclusions differ dramatically in their initial director orientation: for v=1, the director field is aligned, whereas for v=100, the director field is highly distorted and closely follows the shape of the inclusion, see Section 4.1, Fig. 7. Due to a larger orientational mismatch between the director field and the surface in the case v=1, stretch-induced elongation of the inclusion brings about a larger decrease in anisotropic surface energy, and therefore the effective modulus of the composite is smaller for smaller v. In contrast, at small ξ≲0.15, the overall response is close to the response of a composite with isotropic incompressible liquid inclusions and negligible surface tension, as shown in the inset of Fig. 9b. We have verified that these trends remain the same at smaller values of $\omega_{1}$.

We next investigate the effect of mechanical loading on the inclusion shape and orientation of the director field. Fig. 10 shows the inclusion response in the case where v=100 and $\omega_{1}=0.5$, and for two values of ξ. The reference configuration of the droplet, corresponding to $\lambda_{z}=1$, was previously shown in Fig. 7. When ξ=0.01, the inclusion shape changes significantly with the external loading in both tension and compression, while it remains almost unchanged when ξ=10. The director in the inclusion is also more prone to reorientation when ξ=0.01, compared to the case where ξ=10 (Fig. 10, left). This suggests that the distortion of the director field is driven by the shape change of the interface. The scalar order parameter becomes smaller in compression and larger in tension (Fig. 10, right), indicating that not only do the directors become more aligned when the inclusion is elongated, but at a smaller scale, the molecules at each material point also become less dispersed. The case of a smaller anchoring strength ($\omega_{1}=0.1$) but same dimensionless volume (v=100) is illustrated in Fig. 11. The response of the director field under external loading is similar to the case where $\omega_{1}=0.5$, noting that the director and order parameter fields present a larger spatial gradient in the initial state.

**Fig. 13:**
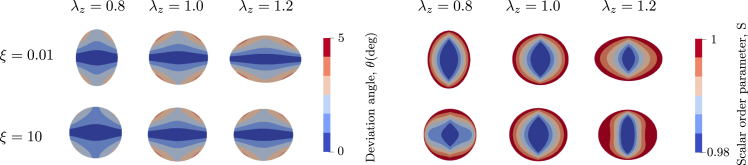
The response of liquid crystal inclusions in a soft matrix under external loading. Contour plots of the director deviation angle θ (left) and scalar order parameter S (right). Material parameters are v=1 and $\omega_{1}=0.1$.

Fig. 12 shows the contour plots of deviation angle and scalar order parameter of inclusions with dimensionless volume v=1 and anchoring strength $\omega_{1}=0.5$. Note the range of the colour bar (0–2.9 deg for deviation angle and 0.98–1 for scalar order parameter), indicating that the molecules are almost aligned in the reference configuration, see also Fig. 7. In contrast to the previous examples, when the dimensionless volume is small, the director field becomes more aligned when the composite is being compressed, while the director field remains almost unchanged in tension. In particular, when ξ=10, although the surface is barely deformed, the deviation angle becomes smaller when the composite is being compressed. We are currently not able to explain this phenomenon. We note, however, that changes in the order parameter field are very small (of the order of 1deg for the deviation angle and 0.01 for the scalar order parameter). Finally, Fig. 13 shows the response of the inclusion for v=1 and $\omega_{1}=0.1$. The effect of elasto-capillary number is the same as in previous results, while the alignment of the director field due to compression is more significant. Similar to Fig. 12, the changes in the absolute values of deviation angle and scalar order parameter are very small compared to the cases shown in Fig. 10, Fig. 11.

## 5 Conclusions

In this work, we have developed a continuum theory for analysing the coupling between elasticity, anisotropic surface tension, and orientational order in composites consisting of nematic liquid crystal inclusions embedded in soft matrices. The theory can also describe liquid crystal inclusions in liquids as a special case. We have implemented our theory in the open-source finite element software FEniCSx, in both 2D and 3D, and conducted parametric studies. Key findings regarding the behaviour of liquid crystal inclusions in soft matrices can be summarised as follows:

• Similar to the case of composites consisting of liquid inclusions in soft matrices, the inclusions reinforce the matrix when the elastocapillary number is large (ξ>1), and weaken the composite when the elastocapillary number is small (ξ<1).
• The reinforcement effect of the liquid crystal inclusion is enhanced by larger anchoring strength, which primarily follows from a composite effect associated with different inclusion aspect ratios in the reference configuration. For a given anchoring strength, the reinforcement effect is reduced when the dimensionless volume is small, due to the alignment of the interface with the director.
• The response of the order parameter field in the inclusion is primarily mediated by the deformation of the interface. Therefore, it is more sensitive to the external loading when the elasto-capillary number is small. Reorientation of the director field is more significant when the dimensionless inclusion volume is large and the anchoring strength is small.

These results are based on the assumption that the shape and order parameter field of the liquid crystal inclusion in the reference configuration of the composite are the same as that of a liquid crystal inclusion with the same properties in a liquid. This may not be exactly the case in experiments, depending on the fabrication method. In addition, results for the composite have been obtained assuming a periodic array of aligned inclusions, loaded along their symmetry axis. Additional numerical studies are need to investigate more general microstructures and loading conditions. However, the continuum theory proposed in this work is general and can be used to simulate other scenarios.

The proposed theory and numerical results are relevant for the design of smart composite materials, for example to optimise the director reorientation under applied mechanical loads. Conversely, the proposed theoretical and numerical methods could be used in conjunction with experimental measurements to estimate material parameters which are difficult to measure directly, for example in natural biological systems. In future work, the theory will be extended to include the couplings with applied electric field. The application of electric field may generate considerable deformation, which could be useful for the design of smart stimuli-responsive materials.

## CRediT authorship contribution statement

**Yifei Bai:** Writing – original draft, Validation, Software, Methodology, Investigation, Formal analysis, Conceptualization. **Laurence Brassart:** Writing – review & editing, Supervision, Methodology, Formal analysis, Conceptualization.

## Declaration of competing interest

The authors declare that they have no known competing financial interests or personal relationships that could have appeared to influence the work reported in this paper.

## Figures and Tables

**Fig. C.14:**
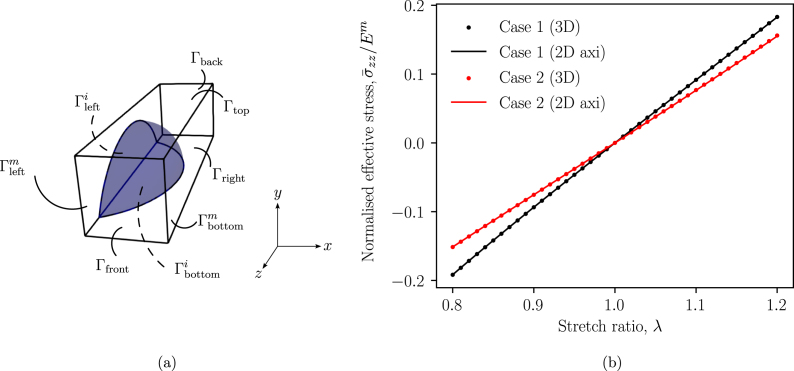
(a) Schematic of 3D unit cell. (b) Comparison between normalised effective stress–stretch relationships obtained using 2D axisymmetric (solid line) and 3D (scatter) UCs.

## Data Availability

We have shared the link to the source code.

## Appendix A Derivation of the weak and strong form

### A.1 Variation δφ

Using Eqs. (12), (14), the variation of the bulk free energy gives:

(A.1) $$\delta_{\varphi }U=\frac{\partial U_{e}}{\partial F}:\delta_{\varphi }F+u_{LdG}\delta_{\varphi }J+J\frac{\partial u_{LdG}}{\partial \text{grad}Q}\;\vdots \;\delta_{\varphi }\text{grad}Q$$

Note the following results:

(A.2) $$\delta_{\varphi }F=\text{Grad}\;\delta \varphi$$

(A.3) $$\delta_{\varphi }f=-f\cdot \delta_{\varphi }F\cdot f$$

(A.4) $$\delta_{\varphi }J=Jf^{T}:\delta_{\varphi }F$$

(A.5) $$\delta_{\varphi }\text{grad}\;d=\text{Grad}\;d\cdot \delta_{\varphi }f$$

 Inserting results (A.2)–(A.5) into (A.1) renders the first term on the left hand side of Eq. (21). Using Eq. (17), the variation of the surface free energy gives:

(A.6) δφU^=u^δφJ^+J^∂u^∂n⋅δφn

Note the following results (Steinmann, 2008):

(A.7) δφF^=Grad^δφ

(A.8) δφJ^=J^f^T:δφF^

(A.9) δφn=−n⋅grad^δφ

 Using the latter result, we can calculate:

(A.10) ∂u^∂n⋅δφn=−[n⊗∂u^∂n⋅i^⋅f^T]:Grad^δφ

Inserting results (A.7)–(A.10) into Eq. (A.6) renders the second term on the left hand side of Eq. (21).

The strong form corresponding to the weak form (21) is obtained as follows. First use integration by parts: (A.11)

∫B0Div(δφ⋅P)−(DivP)⋅δφdV+∫S0Div^(δφ⋅P^)−(Div^P^)⋅δφdS=∫B0B⋅δφdV+∫∂B0T⋅δφdS

Then use the divergence theorem in the bulk and on the interface: (A.12)

∫B0−DivP+B⋅δφdV+∫∂B0P⋅N−T⋅δφdS+∫S0Div^P^+Pi⋅N−Pm⋅N⋅δφdS=0+∫C0(P^⋅N^1+P^⋅N^2)⋅δφdL=0

where $P^{i}$ and $P^{m}$ represent the stress tensor in the inclusion and the matrix, respectively. When using the surface divergence theorem, we have used the fact that P^⋅N=0.

### A.2 Variation δQ

The variation of the bulk energy gives:

(A.13) $$\delta_{Q}U=J\delta_{Q}u_{LdG}=\frac{\partial u_{LdG}}{\partial Q}:\delta Q+\frac{\partial u_{LdG}}{\partial \text{grad}\;Q}\;\vdots \;\text{grad}\;\delta Q$$

The variation of the interfacial energy gives:

(A.14) δQU^=J^∂u^∂Q:δQ

## Appendix B Expression of operators in cylindrical coordinate system

In cylindrical coordinate system with a basis of $\left(e_{r},e_{\theta },e_{z}\right)$, the gradient of an arbitrary vector u w.r.t. the coordinates in the reference configuration with an axisymmetric formulation is:

(B.1) $$\text{Grad}\;u=\left[\begin{array}{ccc} \frac{\partial u_{r}}{\partial R} & 0 & \frac{\partial u_{r}}{\partial Z}\\ 0 & \frac{u_{r}}{R} & 0\\ \frac{\partial u_{z}}{\partial R} & 0 & \frac{\partial u_{z}}{\partial Z} \end{array}\right]$$

The gradient of an arbitrary tensor T is w.r.t. the reference coordinates is:

(B.2) $$\text{Grad}\;T=\left[\begin{array}{ccc} \left[\begin{array}{ccc} \frac{\partial T_{rr}}{\partial R} & 0 & \frac{\partial T_{rz}}{\partial R}\\ 0 & \frac{\partial T_{\theta \theta }}{\partial R} & 0\\ \frac{\partial T_{zr}}{\partial R} & 0 & \frac{\partial T_{zz}}{\partial R} \end{array}\right] & \left[\begin{array}{ccc} 0 & \frac{T_{rr}-T_{\theta \theta }}{R} & 0\\ \frac{T_{rr}-T_{\theta \theta }}{R} & 0 & \frac{T_{rz}}{R}\\ 0 & \frac{T_{rz}}{R} & 0 \end{array}\right] & \left[\begin{array}{ccc} \frac{\partial T_{rr}}{\partial Z} & 0 & \frac{\partial rz}{\partial Z}\\ 0 & \frac{\partial T_{\theta \theta }}{\partial Z} & 0\\ \frac{\partial T_{zr}}{\partial Z} & 0 & \frac{\partial T_{zz}}{\partial Z} \end{array}\right] \end{array}\right]$$

## Appendix C Finite element simulation with 3D unit cell

We have also developed finite element code using a 3D prismatic UC, with dimensions $\left\{2L_{0},2L_{0},2H_{0}\right\}$ in a Cartesian coordinate system. In 3D finite element simulations, we use CG-2 element for displacement field like in axisymmetric simulations, while we use CG-1 element for the pressure field. This is because CG-2-DG-0 combination is not suitable for tetrahedra (Boffi et al., 2013). The volume of the UC is $V=8L_{0}^{2}H_{0}$. Accounting for symmetry, only one quarter of the UC is considered in the numerical simulations (Fig. C.14(a)). In 3D, there are three degrees of freedom to define the tensor Q: two independent components of the director d (the third one is constrained by the condition |d|=1) and the order parameter S. We adopt the following parametrisation of the director:

(C.1) $$d=\cos\theta \cos\psi \;e_{x}+\sin\theta \;e_{y}+\cos\theta \sin\psi \;e_{z}$$

where θ is the angle between the director and the x−z plane, and ψ is the angle between the projection of the director onto the x−z plane and the x-axis. From its definition (11), the order parameter tensor can then be written as:

(C.2) $$\left[Q\right]=S\left[\begin{array}{ccc} \cos^{2}\theta \cos^{2}\psi -\frac{1}{3} & \sin\theta \cos\theta \cos\psi & \cos^{2}\theta \sin\psi \cos\psi \\ \sin\theta \cos\theta \cos\psi & \sin^{2}\psi -\frac{1}{3} & \cos\theta \sin\theta \sin\psi \\ \cos^{2}\theta \sin\psi \cos\psi & \cos\theta \sin\theta \sin\psi & \sin^{2}\theta -\frac{1}{3} \end{array}\right]$$

The boundary conditions for the 3D UC under uniaxial tension and compression write as: (C.3)

$$\varphi_{x}=0\;\text{on}\;\Gamma_{\text{left}}^{i}\cup \Gamma_{\text{left}}^{m}$$

$$\varphi_{y}=0\;\text{on}\;\Gamma_{\text{bottom}}^{i}\cup \Gamma_{\text{bottom}}^{m}$$

$$\varphi_{z}=0\;\text{on}\;\Gamma_{\text{back}}$$

$$\varphi_{z}=2L_{0}\left(\lambda_{z}-1\right)\;\text{on}\;\Gamma_{\text{front}}$$

$$\theta =0,\;\psi =\frac{\pi }{2},\;\frac{\partial S}{\partial n}=0\;\text{on}\;\Gamma_{\text{left}}^{i}\cup \Gamma_{\text{bottom}}^{i}$$

The right and top faces of the UC are constrained to remain flat for periodical boundary condition. This constraint is weakly imposed to the faces by adding the following term to the total potential:

(C.4) $$\int_{\Gamma_{\text{right}}}\left[\beta_{1}\left(\frac{\partial \varphi_{x}}{\partial z}\right)+\beta_{2}\left(\frac{\partial \varphi_{x}}{\partial y}\right)\right]\;dS+\int_{\Gamma_{\text{top}}}\left[\beta_{3}\left(\frac{\partial \varphi_{y}}{\partial x}\right)+\beta_{4}\left(\frac{\partial \varphi_{y}}{\partial z}\right)\right]\;dS$$

where $\beta_{1},\beta_{2},\beta_{3},\beta_{4}$ are Lagrange multipliers defined on the faces.

Fig. C.14(b) shows the normalised effective stress–stretch curves for simulations with 3D UC (dots) and 2D axisymmetric UC (solid lines). Material parameters were the following: Case 1: $\omega_{1}=0.1,v=1$ and ξ=1 (black curves); Case 2: $\omega_{1}=0.2$, v=1 and ξ=0.01 (red curves). ϕ=0.2, γ=0.003 J/${\text{m}}^{2}$, and $L=10^{-8}$ for all cases. Predictions of from both types of UCs are almost identical.

## Footnotes

1 We follow the notation convention used in Steinmann (2008), where Grad(•) and grad(•) denote the gradient with respect to the initial and current configurations, respectively. Similarly, Div(•) and div(•) respectively denote the divergence with respect to the initial and current configurations.
